# Statistical Patterns in Movie Rating Behavior

**DOI:** 10.1371/journal.pone.0136083

**Published:** 2015-08-31

**Authors:** Marlon Ramos, Angelo M. Calvão, Celia Anteneodo

**Affiliations:** 1 Department of Physics, PUC-Rio, Rio de Janeiro, Brazil; 2 National Institute of Science and Technology for Complex Systems, Rio de Janeiro, Brazil; East China University of Science and Technology, CHINA

## Abstract

Currently, users and consumers can review and rate products through online services, which provide huge databases that can be used to explore people’s preferences and unveil behavioral patterns. In this work, we investigate patterns in movie ratings, considering IMDb (the Internet Movie Database), a highly visited site worldwide, as a source. We find that the distribution of votes presents scale-free behavior over several orders of magnitude, with an exponent very close to 3/2, with exponential cutoff. It is remarkable that this pattern emerges independently of movie attributes such as average rating, age and genre, with the exception of a few genres and of high-budget films. These results point to a very general underlying mechanism for the propagation of adoption across potential audiences that is independent of the intrinsic features of a movie and that can be understood through a simple spreading model with mean-field avalanche dynamics.

## Introduction

In recent decades, statistical physics has contributed to the study of social dynamics through theoretical models, providing insights and uncovering the crucial laws that govern many phenomena, such as the spread of information, rumors and opinions [1, 2]. While there has been notable progress in developing theoretical models, their validation by direct confrontation with real data has yet to be achieved. Thanks to the popularization of online social networks and, more recently, of websites for ratings and recommendations, new possibilities have arisen to explore this field. On one hand, ratings provide relevant information on how people’s preferences are distributed. On the other hand, beyond practical applications aiming to improve recommender systems, empirical data allow checking or validating theoretical models that can then be further used to interpret and predict observed outcomes [3].

Here, we aim to explore patterns in movie rating behavior as a source of information on the distribution of people’s preferences. We believe that analyzing the number of votes (where a vote consists of assigning a star rating) rather than, for example, the total number of movie admissions, is a suitable way to measure the popularity of a given movie.

People can watch a movie in many different ways; therefore, the number of admissions provides only partial information. Analyzing a movie’s total gross also suffers from the same problem, and it is difficult to sum all the sources of movie income. Instead, the number of votes is independent of the means used to watch a movie. Furthermore, it is a direct and accessible source of information as well as a much richer one, as we can also extract the public’s opinion about the film. Moreover, while some theoretical models indicate that the distribution of adoption cascades follow a power law [4–7], there is a lack of empirical evidence to validate these results. In this sense, the present work can contribute significantly.

## Empirical results

**Fig 1:**
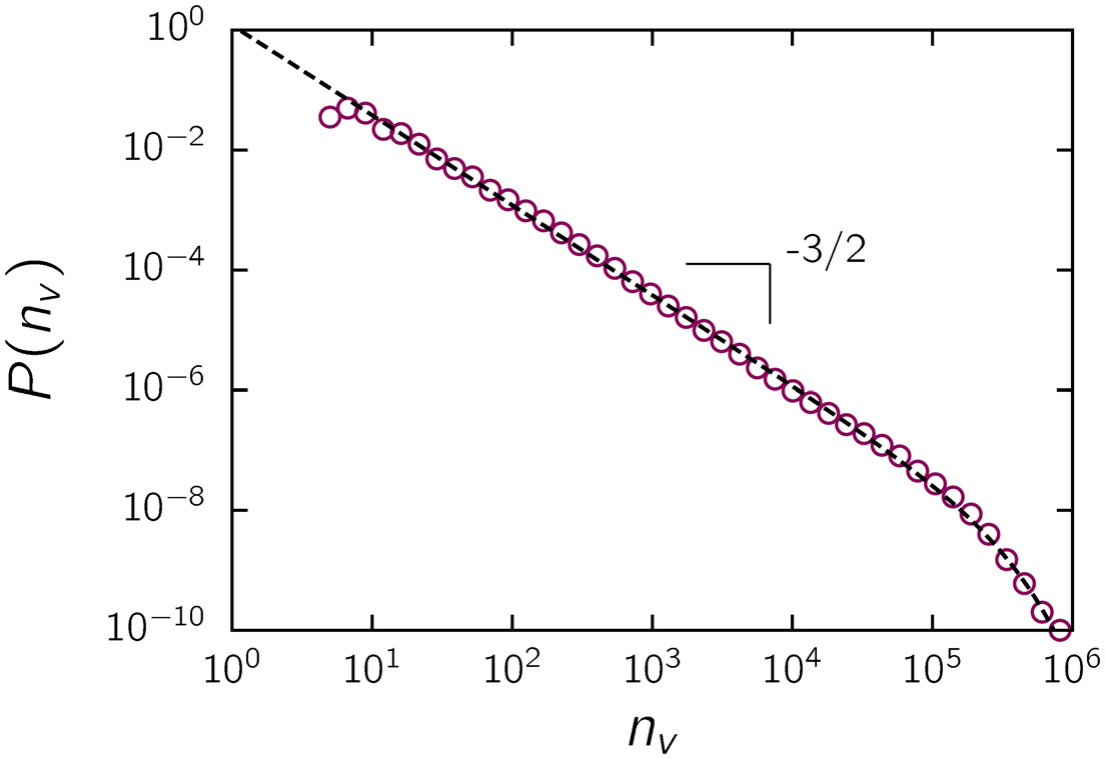
Distribution of the number of votes for IMDb movies (S1 Dataset). The dashed line fits the data of the function $P(n_{v})=An_{v}^{-\alpha }\;\text{exp}(-\lambda n_{v})$, where *A* is a normalization constant, *α* = 1.51 and *λ* = 4.0 10^−6^.

We analyzed data from the Internet Movie Database (IMDb), a source of information about movies and related content that allows visitors to review and rate movies and other entertainment items online (S1 Dataset). It is one of the most visited sites worldwide and the first in its field [8]. We collected the number of votes received by each movie, where a vote consists of assigning a rating on a scale from 1 to 10 stars where 1 star means *awful* and 10 stars means *excellent*. We estimated the probability distribution *P*(*n*_*v*_) of the number of votes *n*_*v*_ by building the normalized histogram shown in Fig 1. A remarkable scale-free behavior over approximately four orders of magnitude of *n*_*v*_ emerges, with a power law exponent 1.51 ± 0.02 and an exponential cutoff. As a test for possible biases, we considered an alternative database provided by Netflix, and we observed the same scaling behavior (In October 2006 Netflix launched a competition to enhance the performance of its recommender system [9]. As part of the competition, they released a dataset that consists of approximately 100 million ratings by users on 17 thousand movies).

There are several processes that can generate power laws [10, 11], in particular, a statistical mixture of different scales. The use of a mixture of processes to explain the scaling behavior is, in fact, a possibility in this case, as the data include different categories of movies, e.g., TV movies, feature films, and items of different ages and with different ratings. Therefore, we should conduct a more refined analysis after separating the subsets by different criteria.

### Rating

**Fig 2:**
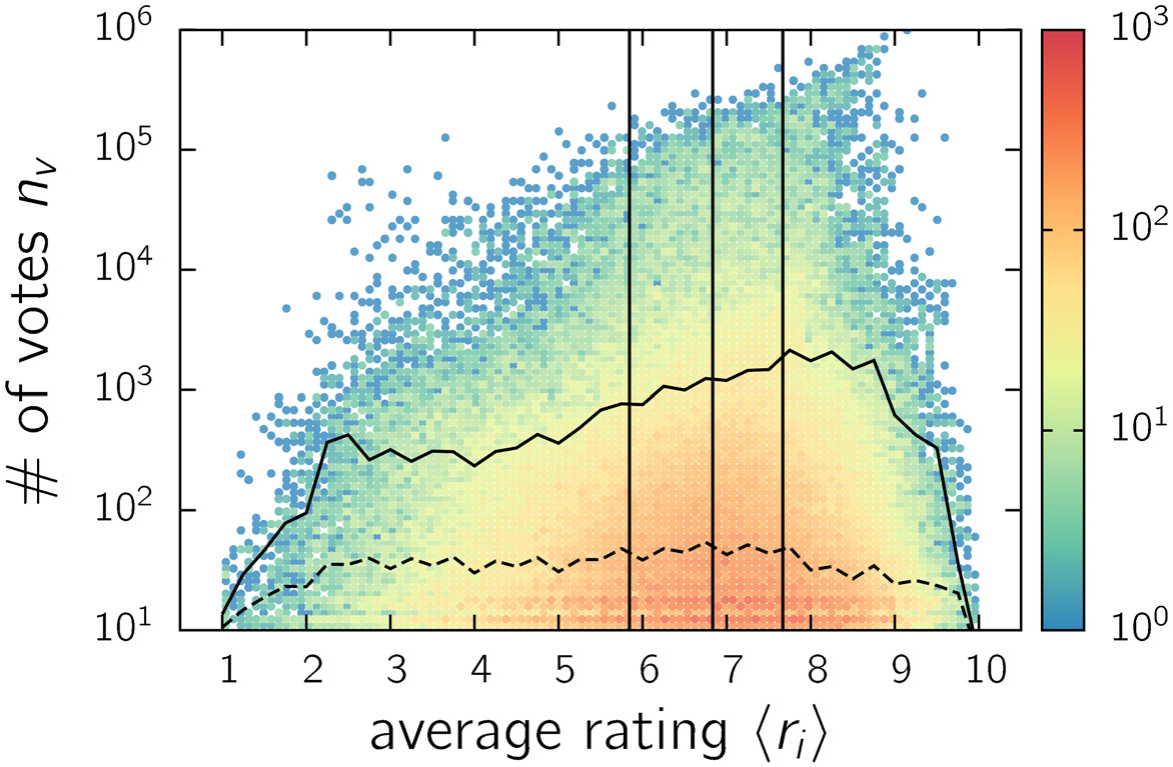
Color map of the number of votes *n*_*i*_ vs. the average rating 〈*r*_*i*_〉 of each IMDb movie *i*. In the color map, each bullet contains the number of movies indicated by the color scale. The white region indicates zero movies. The dotted and dashed lines represent the (binned) arithmetic and geometric mean values, respectively. The vertical lines indicate the quartiles, which divide the dataset into four groups ($G_{4,1}^{r},\ldots ,G_{4,4}^{r})$ of equal size.

We begin by inspecting the relation between the number of votes *n*_*i*_ and the average rating 〈*r*_*i*_〉 of each movie *i*. In Fig 2, we depict a scatter plot of *n*_*i*_ vs. 〈*r*_*i*_〉. Although the points are very spread out, trends can be identified. The logarithmic scale on the ordinate axis implies a broad distribution in the number of votes for each bin of average ratings, as observed for the entire set (Fig 1). The distribution of votes within the range of average ratings is asymmetric, with a bias towards positive values, which differs from the expectations for a random profile. The maximal number of votes increases with the rating, indicating that an extremely large number of votes is associated with well-rated movies. A very similar picture has been reported for ratings on Yahoo music [12, 13].

The number of votes must increase with the number of people that watched a movie, which in turn is expected to be higher when more people like the movie. Moreover, more people liking a movie will drive higher ratings. Therefore, we would expect a positive correlation between ratings and the number of votes. Although the cutoff increases with the average rating, a high number of high-rated movies receive few votes. Moreover, the geometric mean of the number of votes as a function of the average rating presents a flat profile (Fig 2), although the arithmetic mean slightly increases because it is influenced by extreme values.

**Fig 3:**
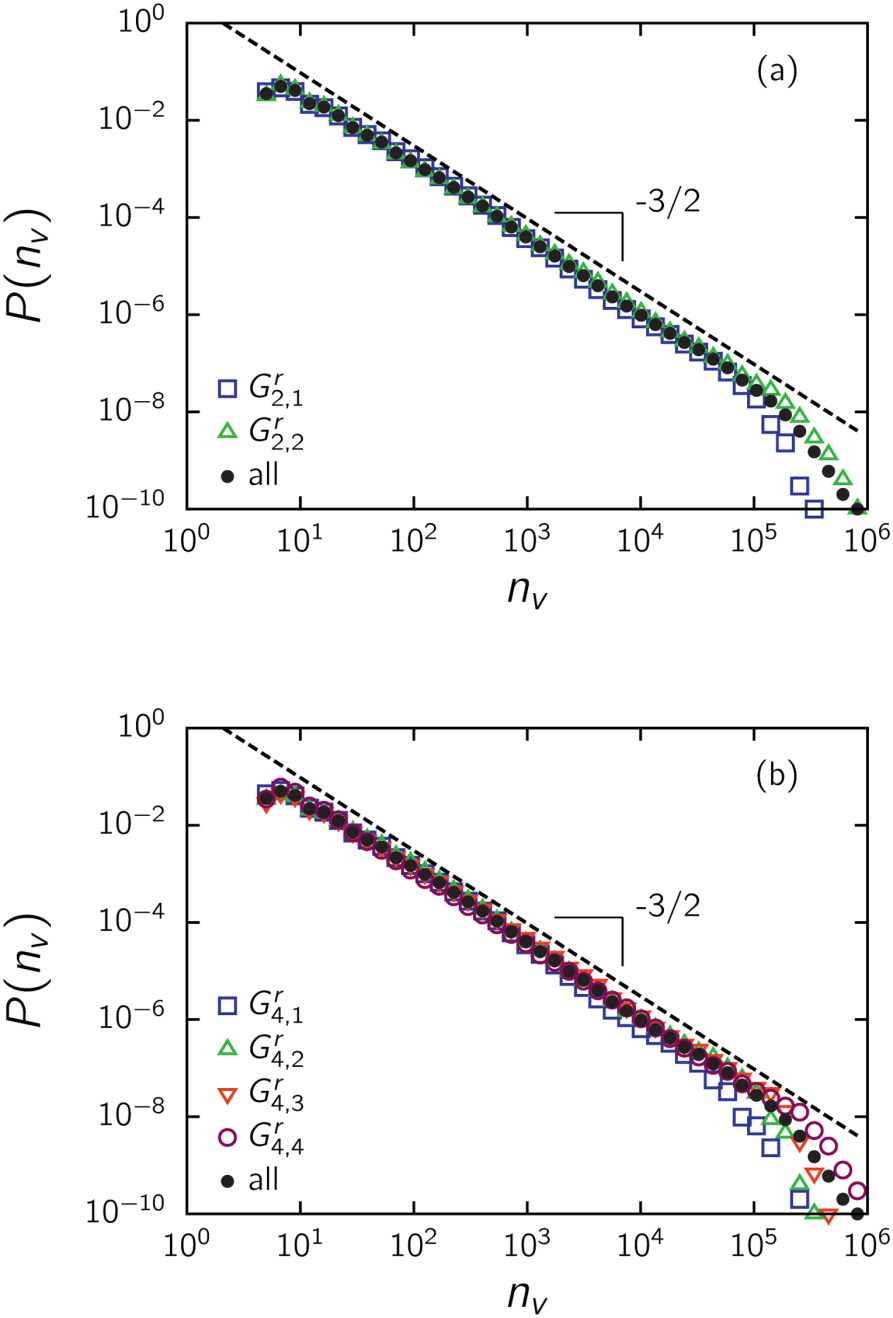
Impact of ratings on the distribution *P*(*n*_*v*_) of votes for IMDb movies, for (a) the two groups $G_{2,1}^{r}$ and $G_{2,2}^{r}$ separated by the median and (b) the four groups $G_{4,1}^{r},\ldots ,G_{4,4}^{r}$ determined by the quartiles, as indicated in Fig 2. In this and other equivalent figures, the dashed line with slope -3/2 is drawn for comparison with the distribution of the entire dataset.

A natural question arises: how is the normalized distribution of votes affected by movie ratings? To examine this issue, we obtained *P*(*n*_*v*_) for separate groups of data, splitting the entire set by the median with respect to the rating. That is, we separately considered the lower- and higher-rated halves of the entire dataset, $G_{2,1}^{r}$ and $G_{2,2}^{r}$, respectively. We also considered the quartiles and subdivided the dataset into the groups $G_{4,1}^{r},\ldots ,G_{4,4}^{r}$. The results are shown in Fig 3. Note that *P*(*n*_*v*_) is almost insensitive to whether a rating is favorable or not. That is, data above and below the median present the same pattern, coinciding over the four decades of the power law regime (Fig 3a). The main discrepancy appears at the exponential cutoff above 10^5^ votes, where the decay occurs faster for the lower-rated half. This is consistent with the fact that low-rated movies do not receive the extremely high number of votes given to high-rated movies. However, the same scale-free behavior holds for both halves over several orders of magnitude in the number of votes, pointing to a mechanism independent of the attributes measured by ratings. The same tendencies are observed at the level of quartiles, as depicted in Fig 3b, and at the level of the number of stars (not shown).

### Year of release

**Fig 4:**
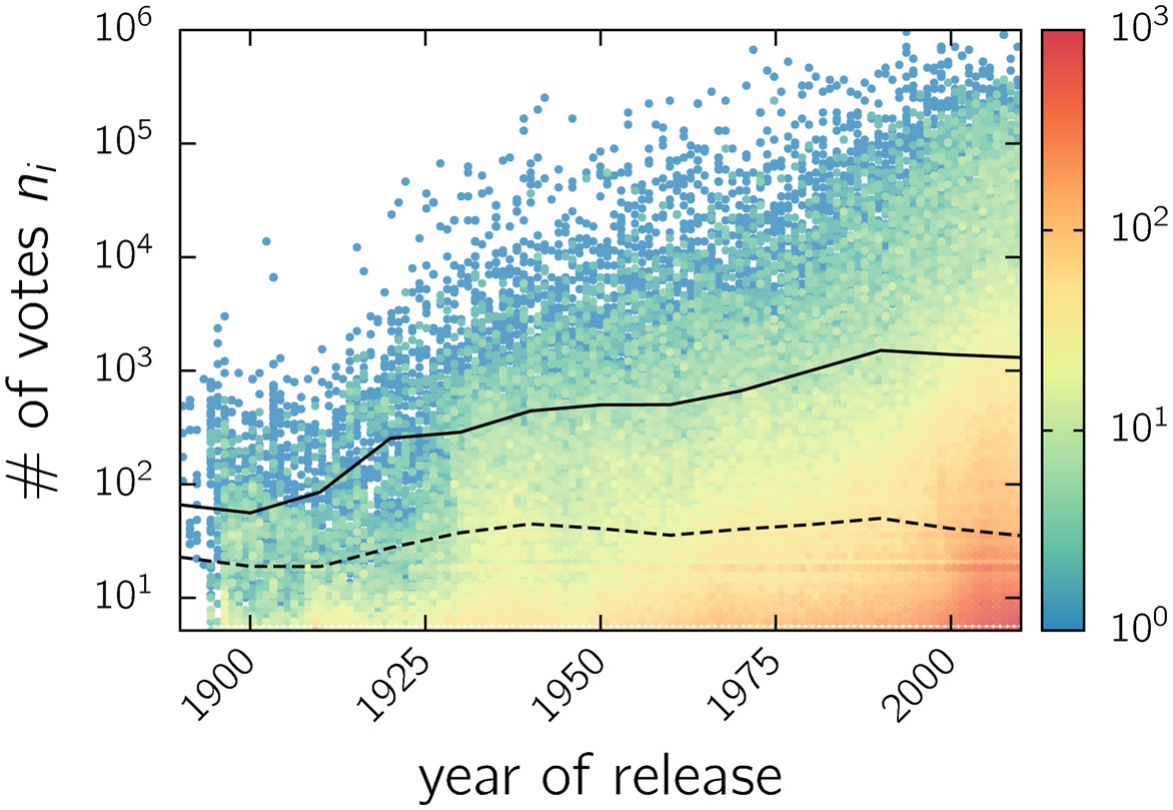
Color map of number of votes *n*_*i*_ vs. the year of release of IMDb movies. Each bullet contains the number of movies indicated by the color scale. The dotted and dashed lines represent the (binned) arithmetic and geometric mean values, respectively.

Second, we investigate the impact of the age of the items on *P*(*n*_*v*_). Fig 4 shows a scatter plot for the number of votes vs. the year of release for each movie. We note that the number of votes depends on the movie’s age. The cutoff for the number of votes increases as age decreases. Concomitantly, younger movies tend to receive more votes on average. All these tendencies are consistent with the expectation that new movies receive substantially more votes than older ones. Furthermore, old movies can receive votes only retroactively (the IMDb user registration system was launched in 1997 [14]), while a new movie can receive votes contemporaneously with its more effervescent phase.

**Fig 5:**
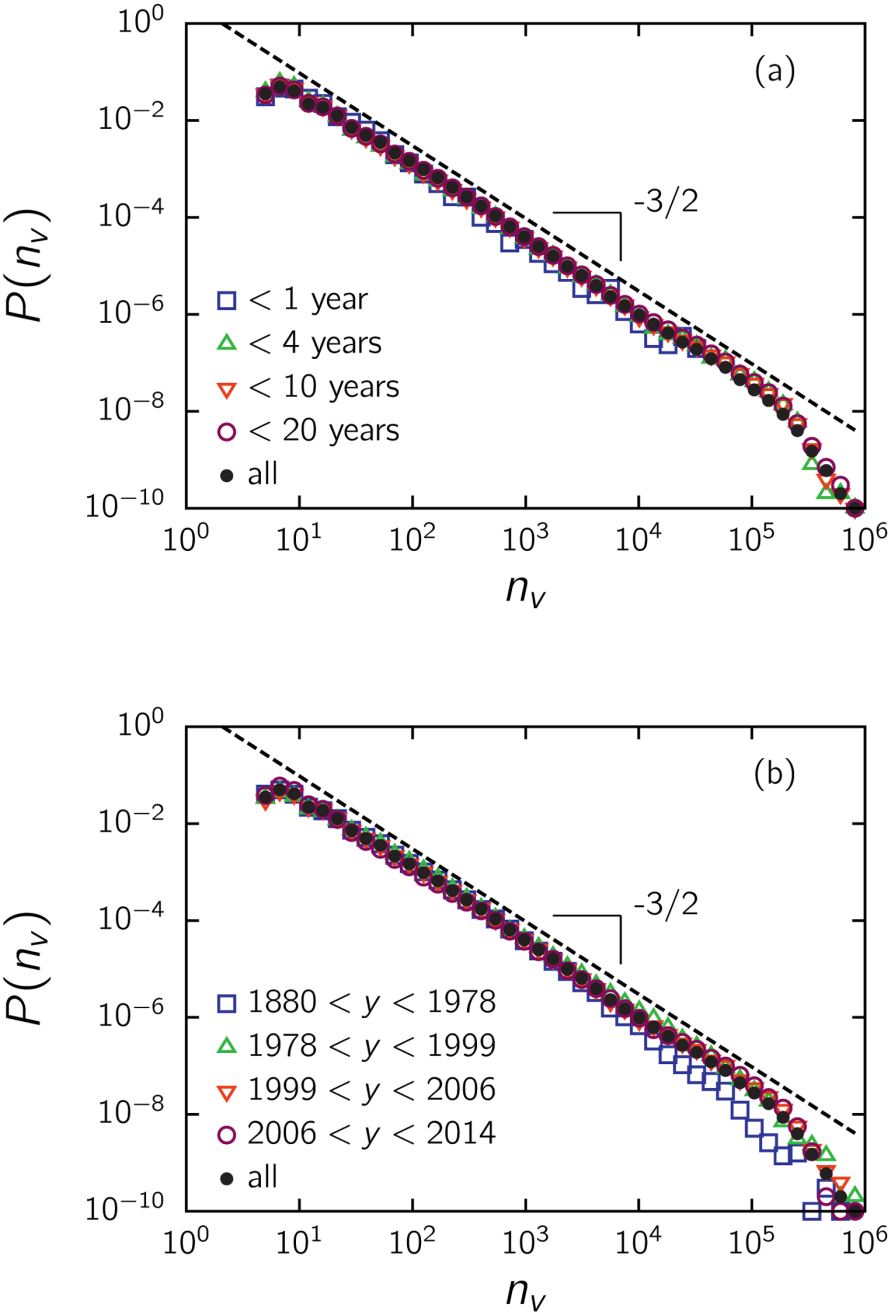
Impact of a movie’s age on the distribution of votes. *P*(*n*_*v*_) for IMDb movies (a) with less than a given number of years, and (b) released within the interval indicated on the figure, chosen to contain the same number of movies.

Again in this case, we analyzed *P*(*n*_*v*_) for subsets separated by the year of release. Sets of movies younger than *y* years, even those released within one year and hence with worse statistics, present the same pattern over the entire interval (see Fig 5a). To take an even closer look, we examined movies grouped by release time interval (Fig 5b). A significant discrepancy exists only for older movies, at the tail above 10^4^ votes, in accordance with Fig 4, which shows that the cutoff occurs at a smaller *n*_*v*_ as age increases. However, the scaling region still spans more than three orders of magnitude of *n*_*v*_, even in this case.

### Genre

**Fig 6:**
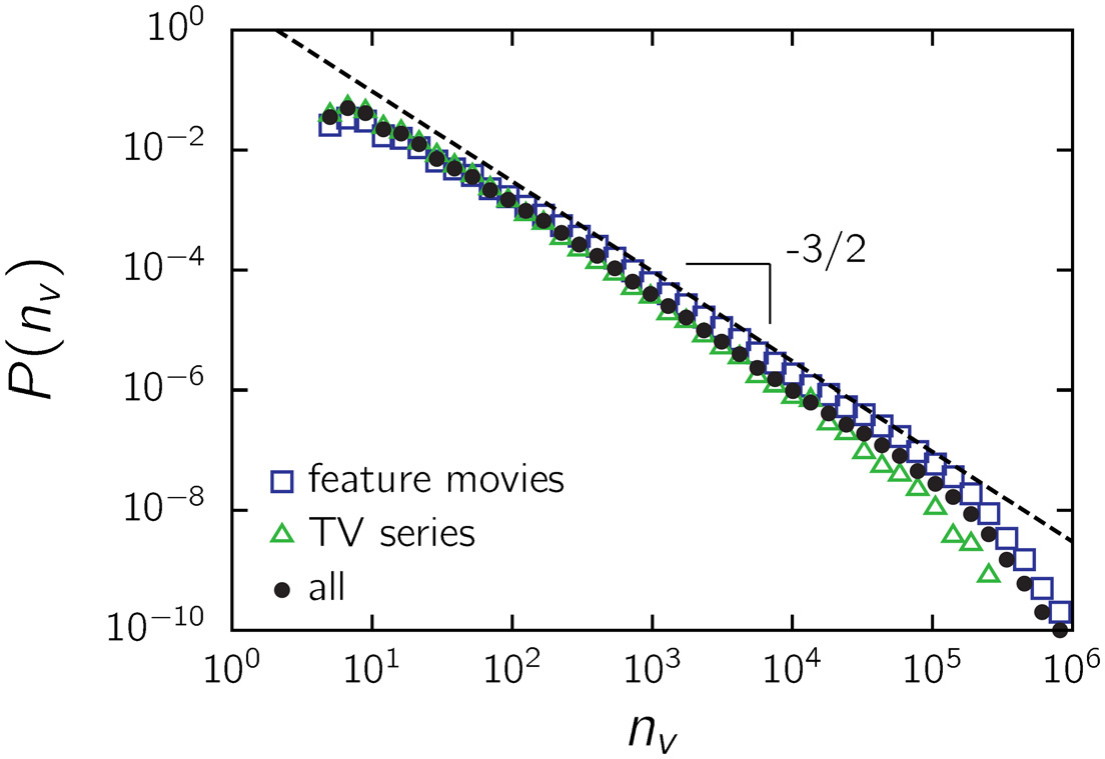
*P*(*n*_*v*_) for TV series and feature movies.

We also show *P*(*n*_*v*_) for TV series and feature movies separately (Fig 6). Both categories behave similar to the entire dataset, with a discrepancy occurring only at the cutoff.

**Fig 7:**
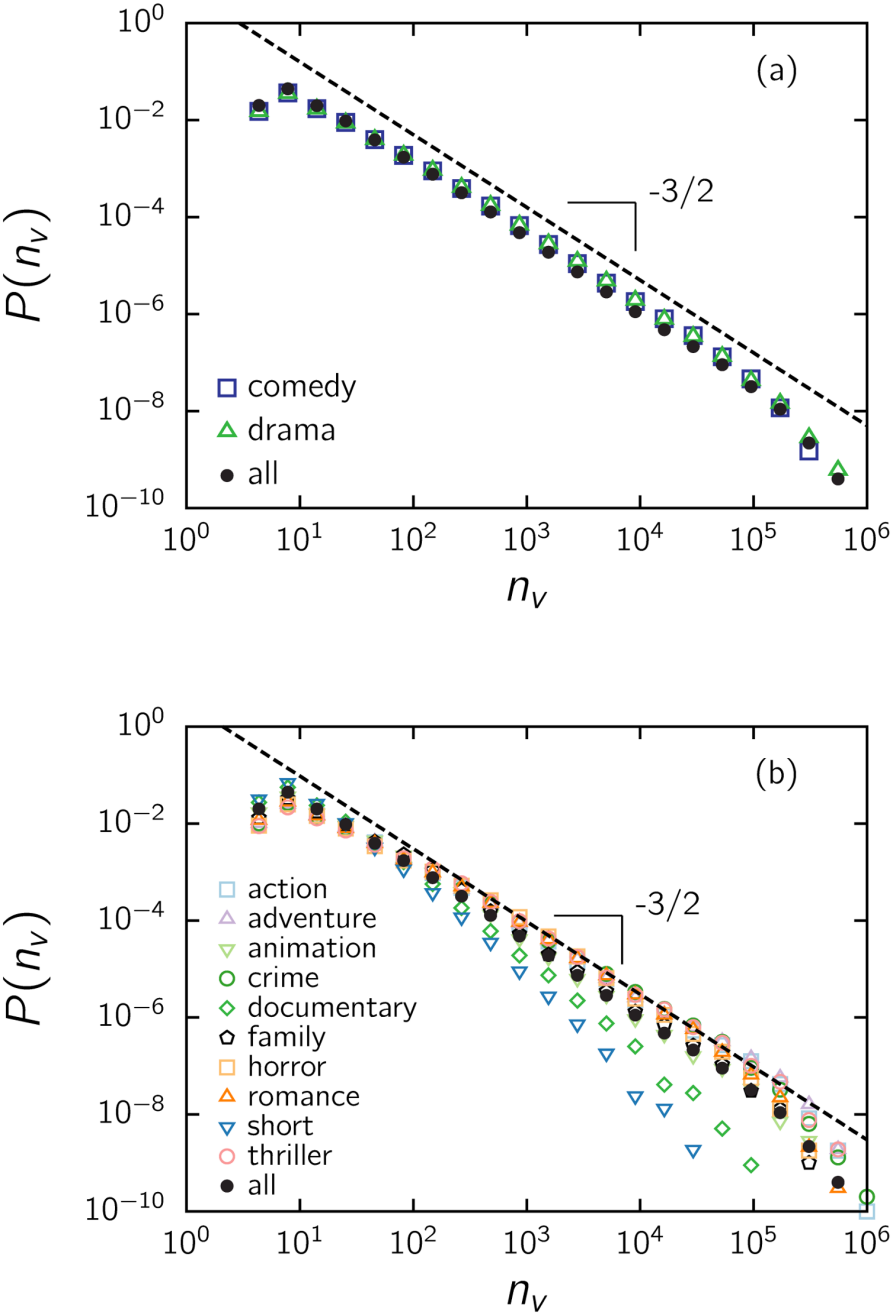
Impact of film genre on the distribution of votes *P*(*n*_*v*_) for (a) dramas and comedies and (b) other genres. Some movies belong to more than one genre.

We further divided the list of items by genre, considering only those genres with more than 15 thousand films (S1 Dataset). The two most numerous genres, comedy (*α* = 1.50 ± 0.03) and drama (*α* = 1.51 ± 0.03), have the same scaling as the entire dataset within error bars and practically coinciding over the entire range (Fig 7a). In addition, the same scaling holds within error bars (±0.04 for the smaller sets) for animation (*α* ≃ 1.56), family (*α* ≃ 1.55), horror (*α* ≃ 1.45) and romance (*α* ≃ 1.46), as shown in Fig 7b. A slightly smaller exponent emerges for action (*α* ≃ 1.42), adventure (*α* ≃ 1.38), crime (*α* ≃ 1.40) and thrillers (*α* ≃ 1.40), with each category having fewer than 20 thousand films (S1 Dataset). Shorts (*α* ≃ 2.1) and documentary (*α* ≃ 1.9) films, which constitute relatively large subsets, present a noticeable discrepancy, with an exponent close to 2. The audiences of these films appear to have a differentiated response.

### Budget

**Fig 8:**
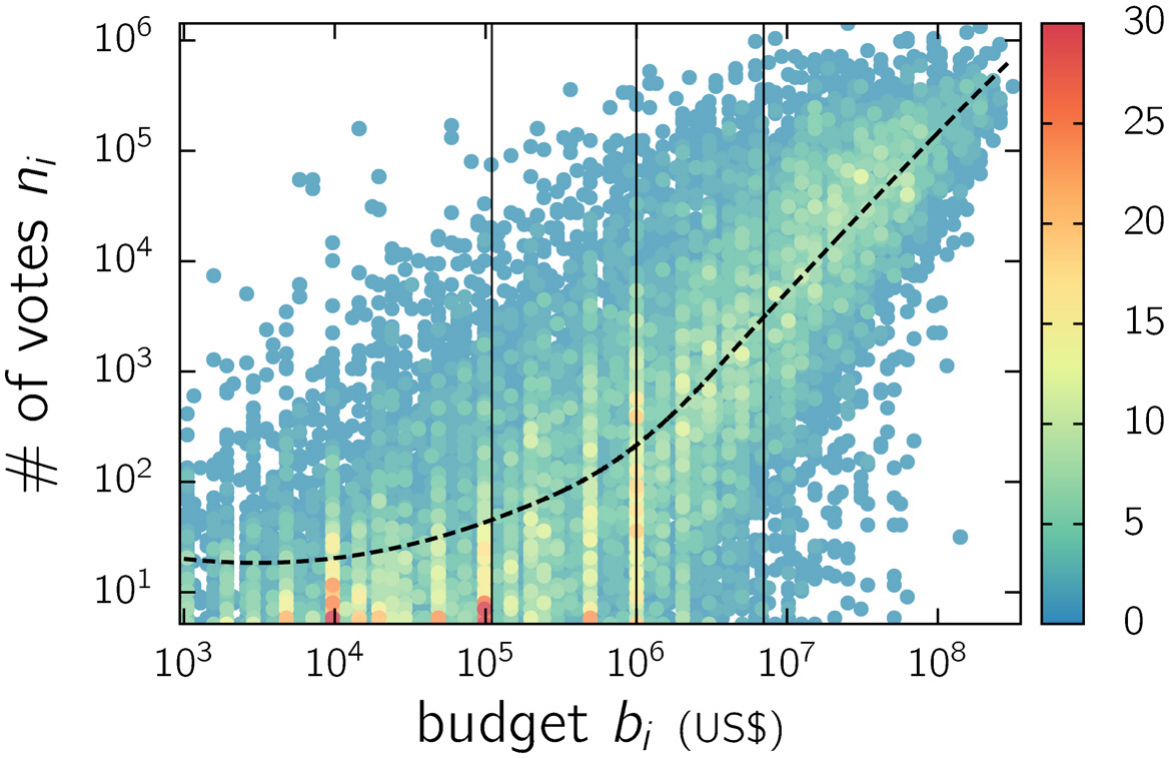
Color map of the number of votes *n*_*i*_ vs. the budget *b*_*i*_ of each IMDb movie *i*. Each bullet contains the number of movies indicated by the color scale. The vertical lines indicate the quartiles. The dashed line was obtained by means of a non-parametric regression rLOESS [15].

Last but not least, we investigated the dependency between the number of votes and the production budget *b*_*i*_ of each movie. Information about budgets is available for a reduced set of feature films, and we considered those with a budget above 10^3^ US$ (approximately 18 thousand films). For this set, we plotted the number of votes vs. budget (Fig 8). This plot shows that points are scattered but display a positive correlation beyond the first quartile, indicating that above a tipping point, on average, the number of votes increases with the budget, although there exist high-budget films with low appeal and low-budget ones with a moderate response.

**Fig 9:**
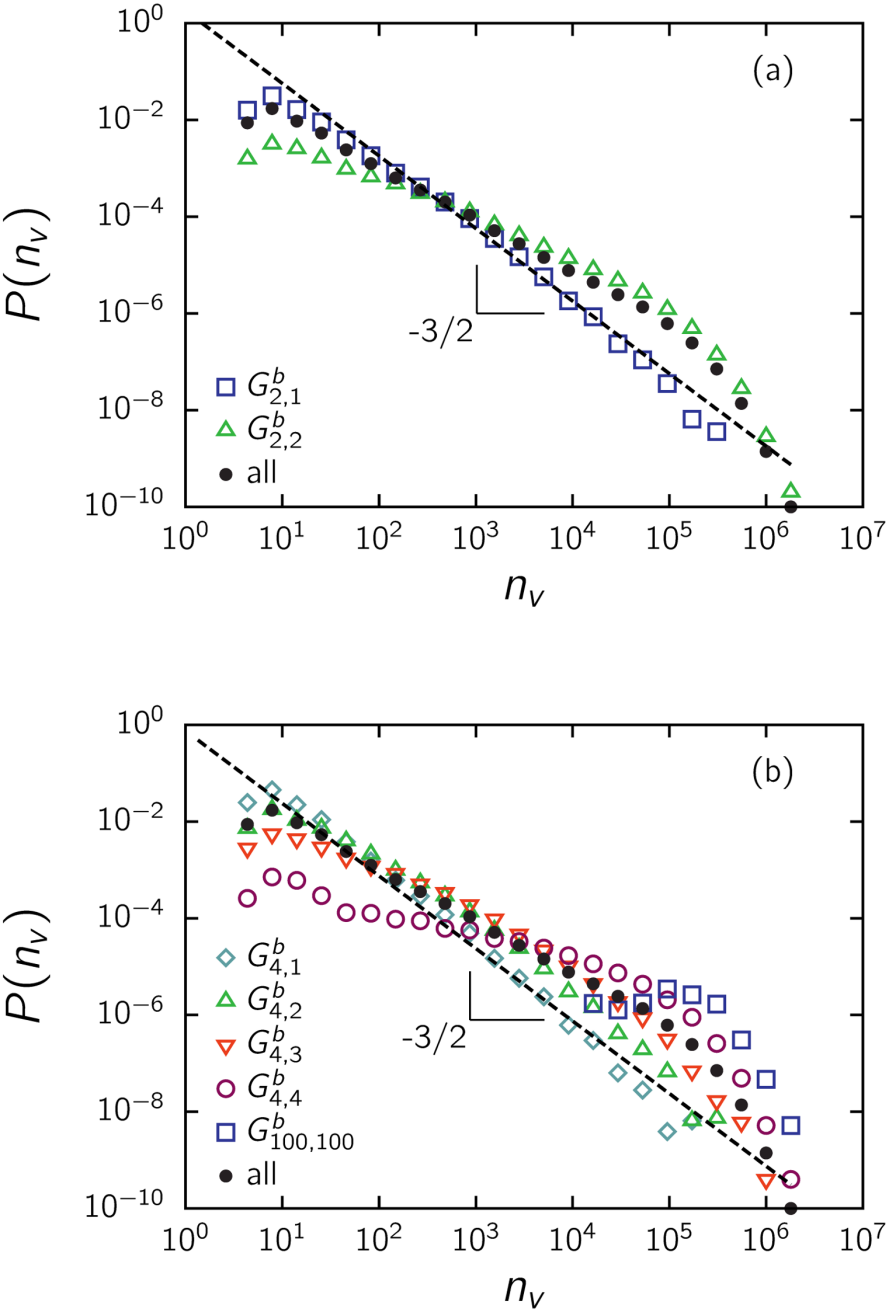
Impact of the budget of feature films on the distribution of votes *P*(*n*_*v*_) for (a) the two groups $G_{2,1}^{b}$ and $G_{2,2}^{b}$ separated by the median with respect to the budget and (b) the four groups $G_{4,1}^{b},\ldots ,G_{4,4}^{b}$ using the quartiles, indicated in Fig 8, and the last percentile $G_{100,100}^{b}$. The dashed line with slope -3/2 is drawn for comparison, as well as the distribution for all films with budget information (only feature films with *b*_*i*_ ≥ 10^3^ US$ were considered).

The distribution of votes for this set (with budget information) does not have the 3/2 power law decay that is characteristic of the entire dataset and shown in previous figures (Fig 9a). To analyze this issue more deeply, we divided the dataset using the median and observed that the high-budget half is responsible for that deviation, while the low-budget half preserves the 3/2 power law. Next, we proceeded to analyze other quantiles. In Fig 9b (and in Fig 8), we see that beyond the level of the median (where the curve given by a non-parametric regression takes off), the distribution completely loses a scaling region. Furthermore, the probability of a large *n*_*v*_ increases with the budget and even develops a peak, as observed for the last percentile. That is, a movie’s production budget practically becomes a determinant of the average number of votes it receives. This result shows that a different generative mechanism governs the distribution of votes given to high-budget films, which is not a surprising result because huge budgets include advertising and publicity actions to reach large audiences. The effect of budget may explain why some genres that are typically associated with high production costs, such as action movies and thrillers, present a slightly smaller exponent than the majority of movies.

## Modeling

People may select movies based on genre, theme, a cast of actors, directors, producers, etc. They are certainly also influenced by publicity and advertising, which are stronger for high-budget movies; however, these movies will be set aside for the moment in the following discussion.

**Fig 10:**
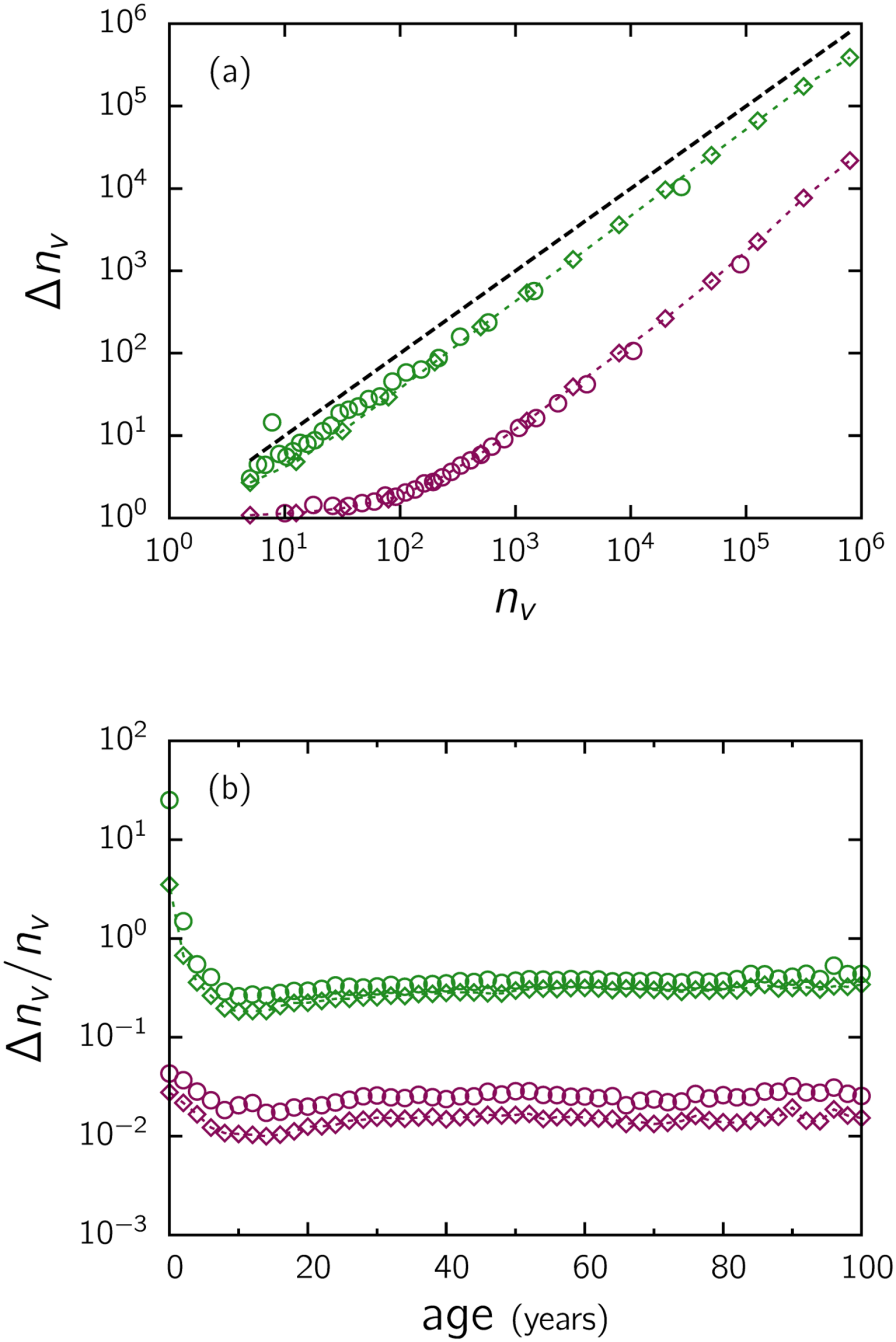
(a) Increment of the number of votes Δ*n*_*v*_ = *n*_*v*_(*t*_2_) − *n*_*v*_(*t*_1_) as a function of *n*_*v*_ and (b) the relative increment Δ*n*_*v*_/*n*_*v*_ = [*n*_*v*_(*t*_2_) − *n*_*v*_(*t*_1_)]/*n*_*v*_(*t*_1_) as a function of the age of the movie for Δ*t* = *t*_2_ − *t*_1_ ≃ 1 month (red, obtained with sets 2 and 3) and 22 months (green, obtained with sets 1 and 2). See (S1 Dataset). The same list of movies, with at least 5 votes at *t*_1_, was considered. The symbols represent the arithmetic (circles) and geometric (diamond) mean values. The dotted lines are a guide; the dashed line in panel (a) with slope 1 was drawn for comparison.

New movies are constantly being released, with actors and directors that are not always sufficiently recognized or popular, and synopses are not always enough to help people decide whether to see a movie or not. Therefore, in many cases, it becomes more practical to adopt other peoples’ opinions [16], through recommendation systems, or suggestions of friends and colleagues, before going to the movies or renting a film. It is common for people choose a movie that someone close to them has watched and commented on, also to avoid feeling excluded in ordinary conversations (fear of missing out [17]). In a more general context, it is known that as more people adopt an item, it becomes more likely that somebody else will want to adopt it [16]. Imitation is a common process in many social scenarios and is very useful as a decision strategy. In fact, in Fig 10a, we observe that the increment Δ*n*_*v*_ = *n*_*v*_(*t*_2_) − *n*_*v*_(*t*_1_) for a given time interval Δ*t* = *t*_2_ − *t*_1_ increases with *n*_*v*_, indicating some type of cumulative advantage.

Because, individuals must decide between two alternatives, to watch or not watch a movie, their state can be characterized by a binary variable, e.g., active or inactive, as considered in many models of opinion contagion [4, 18–21]. To define the rules of contagion, recall that the empirical value of *α* is very close to the standard mean-field value of 3/2 for the avalanche size distribution, as in self-organized criticality with random neighbors [22–24], threshold activation [4] and bootstrap percolation in random networks with finite second moment of the degrees [5–7, 25]. The same scaling also occurs for the cluster size distribution of random networks at percolation [26, 27]. Therefore, the mean-field value suggests some randomness in the dissemination process, independently of the precise pattern of contacts. The present empirical findings indicate that typically, the rules of contagion do not need to incorporate intrinsic features of the movies or of particular audiences, although, as a counterexample, the audiences of short and documentary films appear to behave differently.

Some dissemination rules proposed in the literature require exposure of a node to several active nodes for activation, such as in the threshold model developed by Watts [4], where a minimal fraction of consensus among neighbors is required to infect a node. However, movies can currently be accessed through diverse media, and the choice of one movie does not exclude others; therefore, we believe that in the present case, a single enthusiastic contact may be enough to induce the decision to watch a movie. Therefore, we can consider the simplest case in which a single active node is capable of activating (or infecting) some of its neighbors.

IMDb users represent a sample of the population that watches movies, and, as such, user opinions expressed through the rating system are an indicator of the opinions of the general audience. In particular, the number of votes for a movie is a measure of the general audience’s level of interest in that movie. Therefore, we will make the reasonable assumption that the number of votes is proportional to the number of people that watched the movie.

The full *rating process*, that involves giving a score, is a more complicated process than simply voting. The individual opinion about a movie, recorded in the number of stars, is certainly influenced by social interactions and personal preferences. However, based on the empirical evidences, we can model the statistics of the number of people that became interested in watching a movie, which is manifested in the number of votes, regardless of the scores. This simplification of the full process is motivated by the observation that the main characteristics of the statistics of the number of votes are independent of the score, as well as of other attributes of the movies.

**Fig 11:**
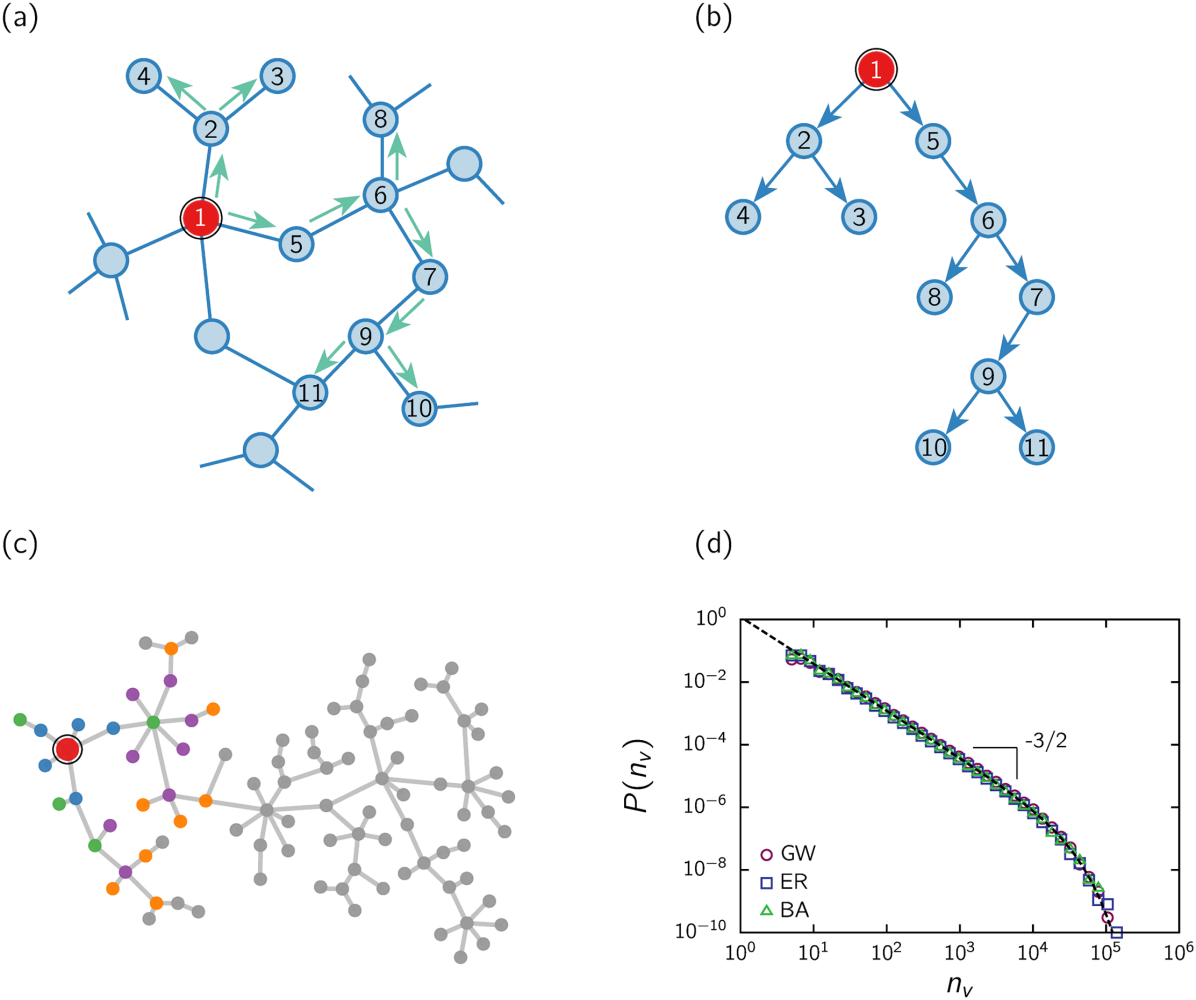
Pictorial representation of the contagion process and the equivalent branching process. (a) Underlying network of contacts. The contagion starts at an initiator node (largest node). Contagion occurs (green arrows) to some of its neighbors (a number of them that we assume to be a random variable) and so on an avalanche develops. (b) A branching tree is built from the contacts that participate of the contagion process. (c) Branching tree realization of a simple Galton-Watson process. The largest node represents the initiator, the first successive generations of the tree are identified with colors, and the final tree is shown as a result of a cascade that becomes extinct at the 13th generation. (d) Distribution of avalanche sizes from simulations of the contagion process: for a simple (network-free) Galton-Watson (GW) process and for the equivalent contagion process on top of Erdős-Rényi (ER) and Barabási-Albert (BA) networks of size 10^6^ and average connectivity 〈*k*〉 = 100. In all cases the probability *p*_*j*_ of influencing *j* individuals was arbitrarily chosen to be exponential with mean *p* ≲ 1.0, and 10^6^ realizations were considered.

In our modeling, one or a few active initiators propagate the idea of watching a given movie, convincing (or infecting) some of their contacts who, in turn, can infect others, and so on. That is, amongst the *k* contacts of an activated node, a random number *j* of them, chosen with probability distribution {*p*_*j*_, with 0 ≤ *j* ≤ *k* < ∞}, becomes activated (see Fig 11a, where we depicted a small connectivity network only for the sake of clearness). The dissemination process stops when, in a given time step, no new nodes become activated. The total number of activated nodes, which we will refer to as an *avalanche* or *cascade*, reflects the number of people who decided to watch the movie.

We simulated this simple contagion process on Erdös-Rényi (ER) [28] and Barabási-Albert (BA) [29] networks, which are representative networks of contacts with homogeneous and scale-free degree distributions, respectively. We assume that the average connectivity 〈*k*〉 is large, like in online social networks (e.g., Twitter, Facebook, etc.) or in real-world relationships. Fig 11d, shows the distribution of avalanche sizes resulting from the simulations, using a discrete exponential probability distribution {*p*_*j*_}, with mean value *p* ≲ 1 (The discrete exponential distribution *P*(*n*) = (1 − e^−*λ*^)e^−*λn*^ was used, with *λ* = 0.7, hence *p* ≡ 〈*n*〉 = e^−*λ*^/(1 − e^−*λ*^) ≃ 0.986). The 3/2 scaling of real data is well reproduced by the model, independent of the network used, as far as the mean connectivity is large enough (〈*k*〉 > > *p*).

The contagion cascade can be *represented* by a branching process: each active individual is represented by a node, and if that individual convinces a contact, then it generates a new branch of the tree; the number *j* of new branches of each node is drawn with the probability distribution {*p*_*j*_}, independent of the process history and of other branches. At a given generation *k* of the branching process, there is a total number of nodes *m*_*k*_. This protocol describes a Galton-Watson (GW) branching process [30]. An avalanche of contagion develops and stops with a certain probability of extinction. Extinction is sure if the mean value *p* < 1.0, otherwise, there is a positive probability of surviving indefinitely. The growth of a tree is analogous to the development of an avalanche in the network. This mapping is illustrated in Fig 11a and 11b. Each movie develops its own independent tree, whose number of nodes reflects the number of people who decided to watch the movie. In Fig 11c, we show a realization in the particular case of the exponential probability distribution {*p*_*j*_}.

The total size of the avalanche *n* = ∑_*k* ≥ 1_ *m*_*k*_ is known to have a distribution *P*(*n*) ∝ *n* ^−3/2^ with an exponential cutoff [30], as depicted in Fig 11d. The cutoff depends on the details of the distribution {*p*_*j*_}, which was chosen in the examples to be exponential, but the exponent 3/2 is a robust result as long as {*p*_*j*_} meets some minimal requirements [30].

The mapping of the network spreading into the GW branching involves the simplification that interferences or overlaps in the network spreading can be ignored, since, in the GW process, branches are independent. When implemented in a network, as the number of infected nodes increases, the contagion probability should decrease, since, in principle, the number of nodes that could be activated diminishes. However, the approximation that the probability of contagion is constant over time is reasonable when the mean connectivity is large, as assumed (〈*k*〉 = 100 in the case of the example). Hence, the distribution of avalanche sizes presents the same scaling, as observed in Fig 11d. Moreover, the outcomes seem independent of the kind of network, which is consistent with the mean-field character of the process.

Despite the simplicity of the model (and the approximations made), it allows to capture the main features of empirical data. It is unnecessary to make any specific assumption about the underling network of contacts, except that the amount of contacts is large enough. Further assumptions are irrelevant to reproduce the 3/2 scaling. However, the categories of short and documentary are counterexamples, displaying a different scaling. In those cases, where the audience is constituted by a singular community, we should have to incorporate more ingredients to the model as will be discussed below.

The type of branching process illustrated in Fig 11 directly maps other models, in particular, the annealed random neighbor version of the sandpile model, a paradigm of self-organized criticality [22–24]. In the random sandpile model, an initiator site topples to its neighbors and a new branch of a tree can be drawn when a neighbor in turn topples, continuing the cascade. In this way, a tree is generated. In that sandpile model, neighbors are randomly chosen at each step so that there is no underlying network of contacts. Even if there is a network of contacts, there are situations, such as when the critical dimensionality is exceeded, where the mean-field character holds. For instance, the mean-field exponent 3/2 is preserved for sandpile models on exponential-type and scale-free networks with a finite second moment. But the exponent increases for very heterogeneous degree distributions [31–34]. Also, for instance, when there is branch overlapping in the dissemination process, the structure of the network of contacts might become important and hence the associated branching process becomes correlated and concomitantly the mean-field character is lost. Something similar may happen for certain audiences, where a different scaling is observed, such as in the case of short and documentary films. In those cases, we should probably need to take into the consideration details of the networks of contacts and/or correlations in the propagation process that can not be neglected. However, such empirical information is not available.

Typical real adoption curves (the number of adoptions as a function of time) for general items, particularly online movies [35], are known to first experience a pre-takeoff stage after release, then grow rapidly in the early stages, reach a peak and finally decay. The integral of an adoption curve gives the size of the cascade. Usually, a real cascade does not become completely extinct, differently to the simulations in Fig 11, but remains at a low level after a certain time because audiences continue to watch old movies. This effect is shown in Fig 10b, where we plot the relative increment Δ*n*_*v*_/*n*_*v*_ = [*n*_*v*_(*t*_2_) − *n*_*v*_(*t*_1_)]/*n*_*v*_(*t*_1_) of the number of votes as a function of the age of the movie (time elapsed since release) for two different time intervals Δ*t* = *t*_2_ − *t*_1_. In both cases, while the increment is large in the initial stage, after a few years, it decays to a low average level.

High-budget films, not considered till now, produce distinct statistics. Hugh budgets imply not only attractiveness stemming from famous actors and expensive settings but also high publicity and advertising budgets. These play the role of an external field that helps to trigger very large cascades. Therefore, it is not surprising that in these cases, the spread mechanism does not only rely on people’s interactions. Instead, without high budgets, interactions could become dominant, and self-organization emerges. Even in this latter case, the power-law scaling indicates that extremely large avalanches are possible, giving rise to blockbusters even among low-budget productions.

## Discussion

A long-tailed distribution of votes, with a power law exponent close to 3/2, emerges from the statistical analysis of IMDb votes. This is a robust result that holds for various subsets defined by movie attributes such as average rating, age or genre, but not for high-budget films. Furthermore, it does not depend on the target audience, suggesting that the dissemination process occurs independently of the pattern of contacts. Genre exceptions are shorts and documentary films, whose audiences show a less heterogeneous pattern.

Remarkably, regardless of whether a movie is “good” enough to be well rated, the scale-free character of the statistics is not affected. The universality of the results indicates that modeling does not require accounting for intrinsic features of the movies or of particular audiences. A type of process that can be naturally associated to the underlying propagation of adoptions is a random multiplicative or branching process, whereby activations (or adoptions) multiply, randomly beginning from a few initial adopters, as illustrated in Fig 11. In this uncorrelated case, cascades emerge with a size distribution decaying as a power law with exponent 3/2. In fact, branching processes are the skeleton of many activation models, producing avalanches in the same universality class [4–7, 25]. As a consequence, the empirical outcomes fit a scenario of imitation cascades. Assuming that voters are a statistically significant sample of the audience, our results represent empirical evidence of this type of cascade.

Meanwhile, high-budget films yield very different statistics, which plausibly reflects that the marketing campaigns associated with large budgets overcome interpersonal activity, acting as an external field that destroys the scaling behavior. Therefore, although the audience–not a movie’s characteristics–makes a movie a hit or a blockbuster [36], large enough budgets can overcome this natural trend.

To evaluate the extent to which IMDb users constitute a biased sample of movie audiences, we considered another similar source, Netflix [9]. We observed the same 3/2 scaling, even though this database contains primarily commercially attractive films, while IMDb is more broad.

The possibility of flaws in the rating system, such as blind voting or massive fan voting, or imprecision in the provided information, cannot be discarded; however, isolated cases are not expected to influence the statistics. Also IMDb is not a static database; the information is constantly being refined by people adding and correcting the data through their “Contributor Zone” section.

Whether other items (consumer goods or cultural products) which can be rated online follow similar propagation processes remains to be investigated. Unlike IMDb, other websites allowing user reviews, e.g., the Apple App Store and Google Play, sell the products being rated, which may mold choices differently. People also choose “items” in political electoral processes. However, as far as the distribution of political votes follows log-normal or power law patterns with different exponents [37–39], the propagation of decisions in that context is of a very different nature because the correlations in the interaction network seem to be relevant. In contrast, in the current case, the dissemination process appears to be uncorrelated. This randomness may be at the root of the difficulties in optimizing recommender systems [3].

Beyond providing a complete explanation for the complex phenomenology related to the spread of adoptions, in this work, we aimed to expand the sources of empirical information related to the field of social dynamics. It is worth remembering that much of the progress in the complex networks field has been driven by empirical observations of real networks. The tools available to rate products can reveal some interesting properties of online social systems, such as the patterns presented in this work.

## Supporting Information

S1 Dataset Description of data from IMDb. We collected votes (from 1 to 10 stars) for all movies, excluding TV episodes (total number of 336,090,882 votes for 300,723 movies), from March 19 to 28, 2013 (set # 1). Using the same list of movies, we collected the number of votes again from December 8 to 18, 2014 (set #2, 465,292,451 votes) and from January 5 to 10, 2015 (set # 3, 471,222,420), as shown in (Fig 10). For budgets, we use a new list and collected data from February 5 to 8, 2015. Results with fewer than 5 votes (in 2013) are not exhibited. Number of items by type: 33,941 (Documentary) 133,775 (Feature Film) 3,172 (Mini-Series) 50,408 (Short Film) 1,071 (TV Episode) 25,168 (TV Movie) 33,165 (TV Series) 2,450 (TV Special) 12,120 (Video) 5,453 (Video Game) By genre: 24,911 (Action); 93 (Adult); 15,651 (Adventure); 18,918 (Animation); 5,385 (Biography); 74,393 (Comedy); 18,693 (Crime); 37,250 (Documentary); 97,087 (Drama); 16,022 (Family); 8,677 (Fantasy); 567 (Film Noir); 1,575 (Game Show); 5,525 (History); 15,072 (Horror); 10,212 (Music); 5,840 (Musical); 8,170 (Mystery); 1,036 (News); 3,605 (Reality TV); 21,165 (Romance); 8,239 (Sci-Fi); 61,538 (Short); 4,360 (Sport); 1,467 (Talk Show); 16,246 (Thriller); 5,080 (War); 4,549 (Western). An item could be defined by more the one genre. As a final observation, it is possible for a user to remove his or her vote; as a consequence, a small fraction of movies have a decreasing number of votes. However, this represents a negligible fraction of the movies. We used the following list: http://www.imdb.com/search/title?title_type=feature,tv_movie,tv_series,tv_special,mini_series,documentary,game,short,video,unknown&user_rating=1.0,10.
(ZIP)
